# Effects of ball type and maturity status on U10 tennis players competition load

**DOI:** 10.3389/fpsyg.2026.1719947

**Published:** 2026-01-16

**Authors:** Manrique Rodríguez-Campos, Ana Piquer-Piquer, José María Giménez-Egido, Jesús Ramón-Llin, José Francisco Guzmán, Bernardino Sánchez-Alcaraz, Goran Vuckovic, Rafael Martínez-Gallego

**Affiliations:** 1 Research Group on Sports Technique and Tactics, University of Valencia, Valencia, Spain; 2 Department of Physical Activity and Sport, University of Murcia, Murcia, Spain; 3 Faculty of Sport, University of Ljubljana, Ljubljana, Slovenia

**Keywords:** biological maturation, external load monitoring, inertial measurement, low-compression balls, work-rest ratio, youth players

## Abstract

**Introduction:**

The use of modified equipment in youth tennis, such as low-compression balls, is widely recommended to optimize learning, participation, and long-term athlete development. However, little is known about the physical demands imposed by these adaptations in under-10 (U10) competitive players, particularly when considering biological maturation.

**Methods:**

This study analyzed 72 simulated tournament matches involving 19 tennis players (10.17 ± 1.1 years; 14 boys, 5 girls). Each player competed under two conditions: green balls (25% lower compression) and standard balls. External load variables (active time, recoveries, and work-rest ratio) were recorded with WIMU Pro™ inertial devices, while covariates such as maturity offset (PHV), years of practice, physical activity level, jump height, and number of tournaments were considered. Bayesian repeated-measures ANOVA models were applied to assess differences.

**Results:**

Model comparisons consistently provided anecdotal to moderate evidence in favor of the null hypothesis for ball type, suggesting that any differences in external load between green and standard balls are likely trivial and of limited practical relevance, with wide credible intervals reflecting substantial individual variability. Conversely, biological maturation (PHV) emerged as the strongest predictor of active time and work-rest ratio, with players closer to their PHV showing reduced active time. Moderate to strong Bayes factors, together with credible intervals excluding zero, indicate a robust and meaningful influence of biological maturation on match demands.

**Discussion:**

These findings suggest that, in U10 tennis, low-compression balls provide a pedagogical advantage without increasing external load. Instead, biological maturation plays a decisive role in modulating match demands. Green balls represent a convenient and developmentally appropriate tool in U10 tennis, but coaches should therefore prioritize maturity-based individualization of training and guiding the transition to standard ball, rather than relying solely on chronological age.

## 1 Introduction

Early dropout from youth sports is a multifactorial challenge, where negative initial experiences play a pivotal role. In this context, adapting the competitive environment, particularly the equipment, has been proposed as a key strategy to favor adherence and skill development, as it shapes both the perceptual–motor demands and the physical effort required during play (Farrow and Reid, 2010; Buszard et al., 2014; Kachel et al., 2015; Limpens et al., 2018). Some systematic reviews reflect how the adaptation of equipment not only responds to biomechanical and physiological criteria, but also to psychological aspects, as it allows children to interact with the game object in a more efficient and meaningful way, promoting motor learning and enjoyment of the activity (Buszard et al., 2016; Chapelle et al., 2023; Piquer-Piquer et al., 2025).

Several studies have shown that these adaptations not only promote players’ physical health, but also improve technique, accuracy, and power of strokes, especially in the early stages of learning (Larson and Guggenheimer, 2013; Prodan and Grosu, 2017; Buszard et al., 2020). In the competitive context, the use of low-compression balls and smaller courts prolong the duration of points, promoting a more dynamic and tactical game, similar to adult tennis, and facilitating the development of advanced skills from an early age (Sánchez-Alcaraz, 2013; Schmidhofer et al., 2014; Kachel et al., 2015; Timmerman et al., 2015; Bayer et al., 2017; Fitzpatrick et al., 2017, 2018; Limpens et al., 2018; Giménez-Egido et al., 2020; Gimenez-Egido et al., 2020a, 2020b; Kilit et al., 2024). However, although longer rallies suggest an increased external load, this relationship has not been objectively quantified in U10 players using green balls.

Adapting the ball is therefore a central element in scaled tennis progressions, even though regulations have established the use of green balls in U10 competitions, with 25% less pressure than standard balls (International Tennis Federation, 2023). There is a critical lack of studies analyzing the physical load during matches for beginner tennis players (under 10) using adapted balls, despite the sport’s high-intensity nature (Crespo and Miley, 1999). Quantifying the competition load is vital for optimizing training and preventing injuries during the formative stages, yet the green-dot ball’s impact remains objectively unaddressed (Soligard et al., 2016; Impellizzeri et al., 2019).

The literature reports several studies analyzing the physical demands of young athletes across different sports using objective monitoring technologies. In physical education settings, variations in internal and external load have been identified in 9-year-old students (Sierra-Ríos et al., 2021). In youth football, Gómez-Carmona et al. (2019b) employed WIMU™ inertial devices to assess external load variables such as total distance, high-intensity distance, accelerations, and Player Load, alongside internal load measured via heart rate in under-19 players. Similarly, Marzano-Felisatti et al. (2024) quantified physical and conditioning demands in under-15 and under-19 beach volleyball players during competition, reporting distance- and acceleration-based variables.

In tennis, analyzing workrest periods is essential to understand the intermittent nature and physiological demands of match play. Previous studies have reported work–rest ratios ranging from 1:3 to 1:5 in professional male players (Kovacs, 2004), 1:2.1 in elite female players on clay courts, with effective playing time representing 21.6 ± 6.1% of total match duration (Fernandez et al., 2006), and 1:2.7 in 15–16-year-old adolescents competing on hard courts, with actual playing time of only 31.50 ± 5.83 min out of a total match duration of 105.00 ± 20.00 min (Torres-Luque et al., 2011). However, despite evidence that rally duration in adolescents is typically below 10 s (9.08 ± 0.60 s), no studies have analyzed work–rest characteristics in younger tennis players.

In relation to the physical demands placed on players who are still growing, it is important to consider the stage of maturity of each individual, as this can affect their performance and physical abilities (Fernandez-Fernandez et al., 2023). Players closer to their PHV typically present higher strength, speed and anaerobic capacity, which may influence the magnitude of the external load they can generate during match play. Maturation status refers to the level or state of maturation at the time of observation and is a non-invasive indicator calculated using the maturity offset. This maturity offset represents the time before or after the peak height velocity (PHV). PHV is the indicator of the moment of maturation, representing the estimated age at which the maximum growth velocity in height occurs during adolescence (Kozieł and Malina, 2018). Therefore, the chronological age at the time of prediction minus the maturity offset provides an estimate of the age at PHV. It is also important to assess the level of sports practice in research with young athletes, so that their level of activity and the relationship between this variable and other characteristics can be understood (Kowalski et al., 1997; Martínez-Gómez et al., 2009).

Given all of the above, monitoring load during sports practice is critical for proper athletic development. Furthermore, technological developments have transformed the way this is assessed, both in sporting and educational contexts. Among these changes, EPTS (Electronic Performance and Tracking Systems) inertial devices have gained prominence as accurate, versatile and accessible tools for analyzing human movement in sport (Gómez-Carmona et al., 2021). These devices allow for the continuous, real-time recording of kinematic parameters such as accelerometry, magnetometry, positioning and tracking, among others. Specifically, GPS (Global Positioning Systems) and inertial devices (IMUs) have been used in team sports to record training load and reduce the risk of injury (Theodoropoulos et al., 2020).

The use of these devices allows for an accurate assessment of movement dynamics during real match play without the need for invasive infrastructure or laboratory-based settings (Gaffney et al., 2009; Whiteside et al., 2017). These devices have been used to assess physical demands in numerous sports: soccer (Boyd et al., 2013; Akenhead and Nassis, 2016; Fernandez et al., 2016; Bayliff et al., 2019), basketball (Espasa-Labrador et al., 2024); padel (Miralles et al., 2025); volleyball (Bellinger et al., 2021; Hank et al., 2024; Marzano-Felisatti et al., 2024); and hockey (Gabbett, 2010), as well as in youth tennis contexts Galé-Ansodi et al. (2017).

Despite scientific support for the use of adapted equipment in children’s tennis, there is a clear lack of studies that objectively analyze the physical demands of these adaptations, especially in players under the age of 10, considering their stage of development. Consequently, a critical disconnect exists between regulatory policy, which is based on assumed pedagogical benefits, and the scientific understanding of its physiological consequences. Clarifying how green balls modulate both external and internal load is therefore essential for guiding evidence-based training in formative stages.

Therefore, the objective of this study was to compare the competition load in U10 tennis players using low-compression balls (green) versus standard balls in simulated tournaments. In addition, we sought to quantify the influence of individual covariates, including biological maturity (PHV), competitive experience, and overall level of physical activity on the recorded demands. Based on previous literature on scaled equipment and youth physiology, we formulated the following hypotheses:

1. We expected that playing with Green balls would lead to greater load compared to standard balls, attributed to the facilitation of longer rallies and fewer errors.
2. We hypothesized that covariates, as biological maturity, would be a positive predictor of external load, expecting players closer to their Peak Height Velocity (PHV) or boys to generate higher absolute physical outputs.

## 2 Materials and methods

### 2.1 Sample

A total of 72 matches with an average duration of 21 min and 56 s (± 8 min and 41 s) were analyzed, involving 19 players (14 boys and 5 girls) aged between 9 and 10 years (10.17 ± 1.1). All participants were competitive junior players, with an average of 3.63 ± 1.45 years of tennis practice and having competed in 7.65 ± 7.36 tournaments on average. Detailed sample characteristics are provided in Table 1.

**Table 1:** Sample description.

	Total (*n* = 19)		Girls (*n* = 5)		Boys (*n* = 14)	
	Mean	S. D.	Mean	S. D.	Mean	S. D.
Age (years)	10.17	1.10	10.5	0.69	10.8	1.23
Height (cm)	138.89	8.46	137.0	71.1	139.6	70.0
Sitting height (cm)	70.21	5.26	70.9	2.51	69.27	5.46
Maturity offset (years)	−2.84	0.98	−2.24	0.87	−3.14	0.84
Peak height velocity (PHV) (years)	12.94	0.95	12.11	0.48	13.26	0.66
Physical Activity Questionnaire (PAQ-A)	3.49	0.57	2.82	0.49	3.77	0.33
Experience in tennis practice (years)	3.63	1.45	4.30	1.20	3.39	1.50
Tournaments played (*n*)	7.65	7.36	2.4	2.51	9.8	5.34
Weekly training hours (*n*)	6.0	2.9	4.8	1.64	6.4	3.17

The players participated voluntarily, providing informed consent signed by their parents prior to the start of the tournament. Throughout the procedure, the ethical principles of the Declaration of Helsinki were followed, and approval of the protocol was obtained from the Ethics Committee of the University of Valencia (2025-FIS-3843453).

### 2.2 Instruments

EPTS (Electronic Performance and Tracking Systems) were used to record information on the external load and performance of the players. Specifically, the device used was the Wimu Pro™ (RealTrack Systems, Almería, Spain), a hybrid EPTS that integrates both Local Positioning System (LPS) technology for indoor facilities and Global Navigation Satellite System (GNSS) technology for outdoor facilities, as well as inertial sensors (IMU) (accelerometers, gyroscopes, magnetometers, barometers) in a single unit (Kim et al., 2023).

**Figure 1:**
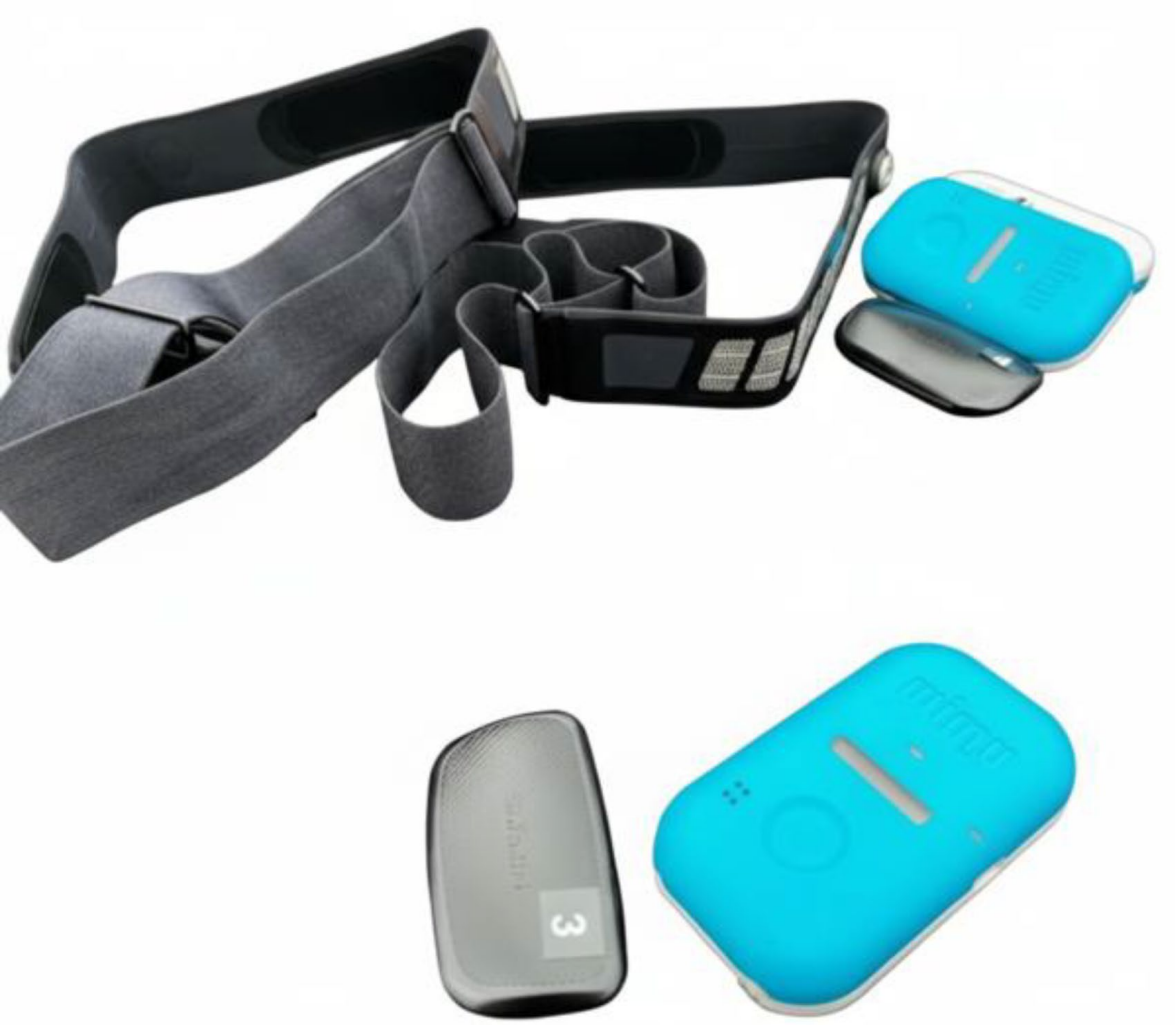
WIMU Pro™ devices used.

Evidence supports that EPTS devices, specifically the Wimu Pro™, are valid and reliable for the objective and detailed quantification of physical load in tennis players and other athletes (Duffield et al., 2010; Hernández-Belmonte et al., 2019). Studies show that both GPS (10 Hz) and UWB (20 Hz) technology are valid and reliable for time-motion analysis in team sports such as football (Bastida Castillo et al., 2018). Triaxial accelerometers (components of IMUs) in particular have been validated for their excellent intra- and inter-device reliability and very high reliability in various anatomical locations, validating their use for calculating external load variables such as Player Load™ or impacts (Gómez-Carmona et al., 2019a). In this study, all data recordings were made on outdoor tracks, using the GNSS system as a receiver, which recorded data at a sampling frequency of 18 Hz, and 1000 Hz for the inertial sensors. During all data collection sessions, the Wimu Pro™ unit was securely placed on the upper back, positioned between the scapulae, using the custom-fit vest provided by the manufacturer (Figure 1).

On the other hand, the maturity status of the players was calculated based on the variables of height and sitting height, following the formulas validated by Kozieł and Malina (2018), which were used to obtain the maturity offset value, presented below:

Girls: Maturity offset (years) = −7.709133 + (0.0042232 X (age X stature)).

Boys: Maturity offset (years) = −8.128741 + (0.0070346 X (age X sitting height)).

Finally, to determine the players’ level of physical activity, the PAQ-A scale (Kowalski et al., 1997) was used, which is a self-administered scale that has been widely used to assess the level of physical activity of schoolchildren over the previous 7 days. In addition, this scale has been validated in Spanish adolescents by Martínez-Gómez et al. (2009). It consists of 8 items scored on a 5-point scale, with an extra item to determine any illness or situation that may have altered their physical activity during that week. To calculate the level of activity, only the average of the other 8 items is calculated.

### 2.3 Procedures

**Figure 2:**
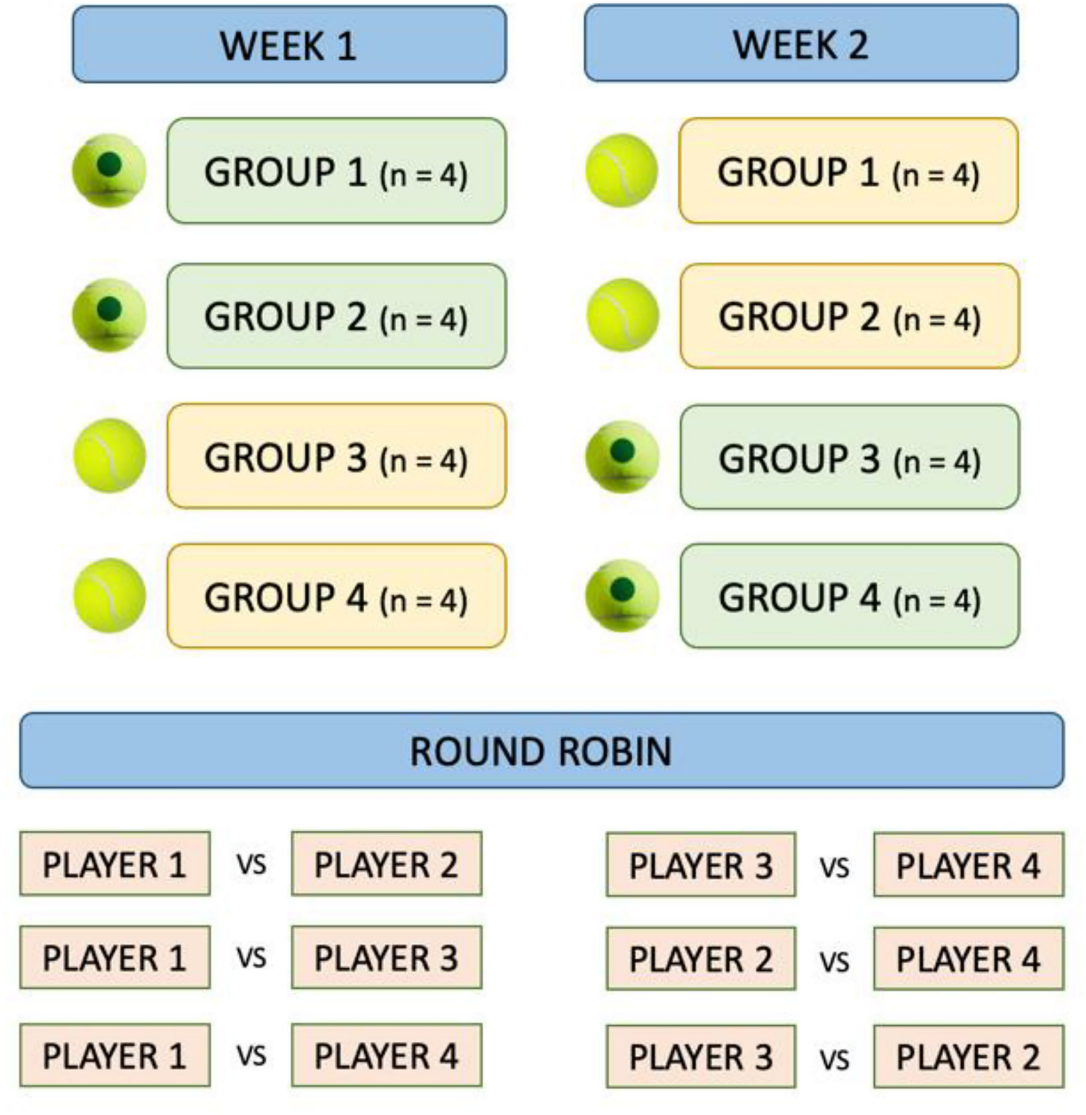
Competition procedure.

A cross-over design was used for the study, with four experimental groups of four players each, so that all participants were exposed to both conditions, allowing comparisons within the same groups in different situations. Participants were allocated to these groups based on their skill level to ensure balanced competitive levels within each round-robin group. Due to the limited availability of official rankings in this age group, participants were allocated to these groups by certified coaches, based on skill level and years of practice. This stratification was intended to avoid mismatched pairings and guarantee representative physiological and locomotor demands during matches. The matches were played in groups in a round-robin format, with one set of four games with a golden point, to avoid time differences that would excessively exceed the duration of the competition and the break times between matches. The competition took place on two different days, ensuring that all participants were exposed to both conditions, with two groups playing with the green ball (with 25% less pressure) and the other two groups playing with the standard ball, and the following week in reverse order, as shown in Figure 2.

Prior to a 5 min on-court warm-up, the players’ height and seated height were measured to the nearest 0.1 cm with a tape measure on a flat vertical surface. For the standing height, participants stood barefoot with their heels, buttocks, and upper back in contact with the vertical surface and tape measure. For the sitting height, participants were seated on the floor with their back and buttocks touching the vertical backboard. The head was positioned in the Frankfort plane (orbitale-tragion line horizontal), and traction was applied to the mastoid processes to ensure full spinal extension before measurement (Hawkes et al., 2020). These metrics were subsequently used to calculate leg length and maturity offset.

The players were then fitted with heart rate bands and tracking vests with the Wimu Pro™ device activated. New balls were used in each of the groups and all the matches were played in hard greenset courts. Data was recorded from the start of the matches with the player’s first serve until the end of the match. After the matches, the data was analyzed using SPro software (version 2.3.1) via the Intervals Pro monitor, adapting the speed values to the players’ ages.

### 2.4 Variables

Wimu Pro™ devices recorded more than 250 variables, of which the most relevant in terms of active times and rest times were analyzed. The external load variables have been defined according to Hudl WIMU Support (n.d.) and were processed using SPro software (RealTrack Systems, Almería, Spain).

#### 2.4.1 Dependent variables

The temporal structure of the matches was analyzed using variables derived from the raw triaxial accelerometer data (ACELT). The active times and recoveries are metrics directly obtained from the SPro algorithm, which identifies active movement intervals through a threshold of 0.02 a.u. in the intensity of the Fast Fourier Transform (applied to the total acceleration data). This processing protocol aligns with the methodological procedures using this technology Oliva-Lozano et al. (2023). Based on this, the following variables were obtained from each match:

- *Active time (s)*: The total time, in seconds, during which the FFT intensity of the ACELT signal was above the 0.02 threshold. This variable represents the cumulative duration of all work periods.
- *Recoveries (s)*: The total time, in seconds, during which the FFT intensity of the ACELT signal was at or below the 0.02 threshold. This variable represents the cumulative duration of all recovery periods between efforts.
- *Active times (cnt)*: The total number of discrete active intervals. A new interval was counted each time the signal intensity crossed from below to above the 0.02 threshold.
- *Work-rest ratio*: A dimensionless value calculated by dividing the total active time (s) by the total recoveries (s). It serves as a key indicator of the intermittent nature of the competition.

#### 2.4.2 Independent variable

*Ball type*: green (25% less pressure) versus standard (International Tennis Federation, 2023; RFET and CEAT, 2025).

#### 2.4.3 Covariate

The following were considered as covariates: peak height velocity (PHV), estimated using the maturity offset equations of Kozieł and Malina (2018); general physical activity level, using the PAQ-A questionnaire, validated in the adolescent population and in its Spanish version (Kowalski et al., 1997; Martínez-Gómez et al., 2009), years of tennis practice (Years of Practice) as an indicator of experience in the sport, jump height (Jump Height) as an indicator of explosive capacity, and the number of tournaments played (Tournaments). These covariates made it possible to control for possible effects of development, physical condition, and experience on the dependent variables.

### 2.5 Data analysis

The analysis began with an exploratory phase of the data, checking the distribution of the data using the Shapiro Wilks test and visual inspection of the Q-Q plots for residuals, assuming normality when the null hypothesis was not rejected and when the observed distribution showed no systematic deviations in the graph. The descriptive comparative analysis of the intrasubject dependent variable was calculated using means, standard deviations, and 95% credibility intervals (95% CrI). A Bayesian repeated measures ANOVA was chosen for the inferential analysis due to its advantages in quantifying evidence for both the alternative and null hypotheses and its robustness with complex models including random effects (van Doorn et al., 2021). The design included Ball (Green, Standard) as an intra-subject factor and considered a random intercept per subject with random slopes for repeated factors, in line with the recommendations for specifying maximum mixed effects models (van Doorn et al., 2021).

Likewise, PH, PAQ-A, Years of practice, Jump Height and Tournaments were explored as covariates, following an approach of comparing alternative models with the null model and analyzing the inclusion of main effects. Although some covariates may reflect related aspects of experience (years of practice, tournaments played, and PAQ-A), they were retained simultaneously to represent distinct conceptual dimensions, such as training exposure, competitive experience, and habitual physical activity. In this context, the Bayesian framework using model averaging naturally accounts for shared information among predictors by penalizing redundant covariates, which is reflected in their posterior inclusion probabilities. Given that the total number of estimated models was high (64 in total), the results only present the five models with the highest relative support according to the Bayes factor (BF10), in order to facilitate interpretation and focus the analysis on the structures with the most evidence from the data. In addition, classic Bayes Factors were estimated for opposing hypotheses (H₀ vs. H₁), interpreting BF₁₀ as the evidence in favor of the alternative hypothesis versus the null hypothesis, and BF₀₁ as its reciprocal (Berger, 2006; Gallistel, 2009; van Doorn et al., 2021). In this way, the Bayes Factor was used as a continuous measure of evidence that quantifies the relative predictive performance between the two hypotheses, also known as marginal likelihood (Etz et al., 2018).

The qualitative interpretation BF10 to determine the effect was performed following the scale of Lee and Wagenmakers (2013) based on Jeffreys (1961): BF < 1/100: extreme evidence in favor of H₀ (exclusion of the effect); BF from 1/100 to 1/30: very strong evidence in favor of H₀; BF from 1/30 to 1/10: strong evidence in favor of H₀; BF from 1/10 to 1/3: moderate evidence in favor of H₀; BF10 from 1/3 to 1: anecdotal evidence in favor of H₀; BF from 1 to 3: anecdotal evidence in favor of H₁; BF from 3 to 10: moderate evidence in favor of H₁; BF from 10 to 30: strong evidence in favor of H₁; BF from 30 to 100: very strong evidence in favor of H₁; BF > 100: extreme evidence in favor of H₁. For each main effect, Bayesian Inclusion Factors (BF_incl) were estimated, which compare the predictive performance between models containing the effect and equivalent models without that effect. In practical terms, BF_incl quantifies how much the probabilities in favor of an effect being present change after observing the data, compared to the prior probability (Rouder et al., 2012; van Doorn et al., 2021).

In the case of BF_incl, the usual criterion was applied, whereby values > 3 support the inclusion of the effect, values < 1/3 support its exclusion, and values between 1/3 and 3 are interpreted as anecdotal (Jarosz and Wiley, 2014; Wetzels et al., 2011). The relative error (%) accompanied each estimate of the Bayes factor and the BF incl as a measure of the stability of the calculation algorithm. According to the recommendations (van den Bergh et al., 2023; van Doorn et al., 2021), an error of < 3% was established as an acceptable criterion, although values close to 0 (< 1%) were considered indicators of a robust estimate. In addition to evaluating the coefficients of determination (R^2^) as a measure of overall model fit, a sensitivity analysis was performed by restricting the comparison to only those variables that showed evidence of inclusion. This strategy allows for the evaluation of the robustness of inferences when models are limited to predictors with greater empirical support, in line with previous methodological proposals (Fernández et al., 2001). Thus, the selective inclusion of variables with empirical support not only facilitates interpretation but also constitutes an additional verification of the stability of the findings.

Finally, for the intra-subject Ball factor, Bayesian *post hoc* tests were performed, directly comparing the levels of the factor. These did not conform to the Bayesian correction for multiple comparisons proposed by Westfall et al. (1997) since there were only two levels (Green vs. Standard). Individual comparisons were performed using the default Bayesian t-test, using a Cauchy *a priori* distribution (0, r = 1/√2). The calculation of specific Bayes Factors (BF₁₀) for the pairwise contrast was performed with the same qualitative interpretation described above. Statistical analyses were calculated using a Bayesian repeated measures ANOVA in JASP software (version 0.95.0.0; JASP Team, 2025). The results were reported following the guidelines proposed for the application and reporting of this type of procedure (Kruschke, 2021; van Doorn et al., 2021).

## 3 Results

The results are structured by dependent variable, with model comparisons and evidence of inclusion effects presented in tables. In addition, comparisons of dependent variables based on ball type are presented in illustrations.

### 3.1 Active times (cnt)

Table 2 shows the comparison of the alternative models with the null model. The best model with predictors was PHV (BF₁₀ = 1.122), followed by PHV + Years of Practice (BF₁₀ = 0.743) and Years of Practice (BF₁₀ = 0.666). Overall, the relative likelihood ratios are very close to 1, so the evidence is anecdotal. The average R^2^ for the models was 0.238 (95% CrI = [0.130, 0.377]). In this regard, the sensitivity analysis including covariates with BF_incl > 1 (PHV) indicated an R^2^ of 0.211 [0.115, 0.339], maintaining the model fit.

**Table 2:** Comparison of Bayesian models with random effects per subject (top 5 of 64) for the variable active time (cnt).

	P(M)	P(M|data)	BFM	BF10	Error %
Null model (incl. subject and random slopes)	0.016	0.078	5.312	1.000	
(PHV)	0.016	0.087	6.021	1.122	1.228
PHV + Years of practice	0.016	0.058	3.862	0.743	0.778
Years of practice	0.016	0.052	3.440	0.666	1.147
PHV + PAQ-A + Years of practice	0.016	0.038	2.500	0.491	4.051

P(M), prior model probability; P(M|data), posterior model probability; BFM, Bayes factor; for the model; BF10, Bayes factor (Model 1 vs. Model 0); PHV, Peak height velocity; PAQ-A, general physical activity level.

The inclusion analysis presented in Table 3 showed anecdotal evidence in favor of PHV (BF_incl = 1.337). The rest of the effects presented BF_incl < 1 (Ball, PAQ-A, Jump Height, Years of Practice, Tournaments), suggesting a lack of support for their inclusion with the current data. The subsequent model-averaged summary indicated a stable intercept (*M* = 51.403, SD = 2.558; 95% CrI = [46.199, 56.436]). For the predictors, all 95% CrIs included zero, indicating uncertainty about their point influence:

**Table 3:** Evidence of inclusion of effects (BFincl) and confidence interval of the averaged posterior summary in the Bayesian ANOVA of repeated measures for the covariates and the factor explaining the variability of active time (s).

	95% CrI posterior	P(incl|data)	P(excl|data)	BFincl
Ball				
Green	[−4.36, 3.21]	0.200	0.800	0.249
Stand.	[−3.39, 4.12]			
PHV	[−8.79, 0.93]	0.572	0.428	1.337
PAQ-A	[−13.10, 8.36]	0.332	0.668	0.498
Jump Height	[−1.68, 2.09]	0.308	0.692	0.446
Years of Practice	[−5.12, 1.11]	0.448	0.552	0.813
Tournaments	[−0.74, 0.59]	0.301	0.699	0.431

CrI, credibility interval; P(incl|data), posterior inclusion probability; P(excl|data), posterior exclusion probability; BF_inl_, Bayes factor for inclusion; PHV, peak height velocity; PAQ-A, general physical activity level.

**Figure 3:**
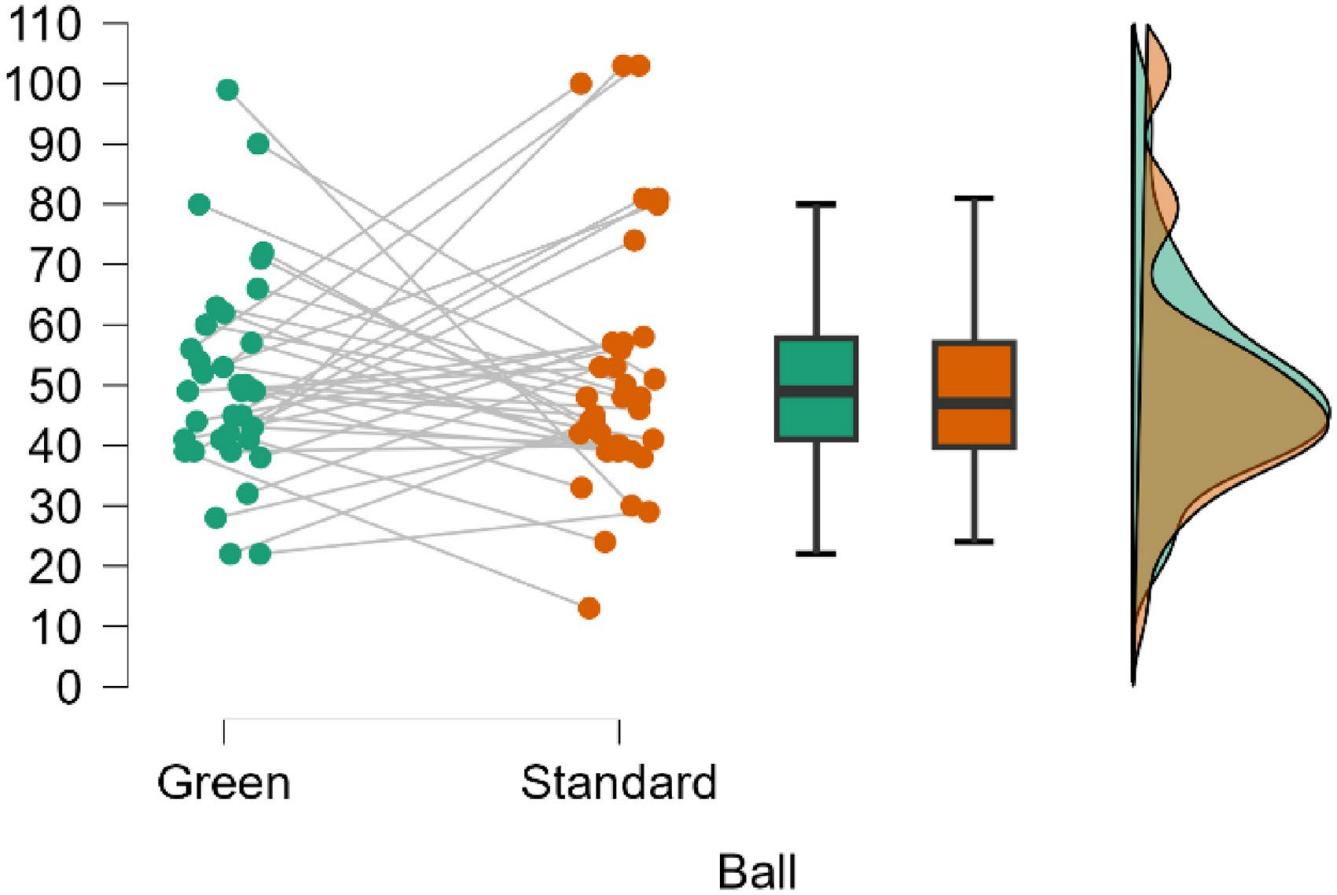
Comparison of the variable active time (cnt) according to the ball used in the match.

The means by condition (ball) shown in Figure 3 were similar and overlapped considerably: Green (*M* = 50.89, SD = 16.83; 95% CrI = [45.20, 56.58]) and Standard (*M* = 51.92, SD = 21.26; 95% CrI = [44.72, 59.11]). The post hoc comparison of Green vs. Standard showed posterior odds equivalent to a BF₁₀, U = 0.183, implying greater support for the null hypothesis (BF10 ≈ 0.183). In practical terms, the data favor the absence of a difference between balls approximately 5.5 times more than the alternative. The percentage error in the estimation of the Bayes factor was 0.044%.

### 3.2 Active times (s)

The best model, as shown in Table 4, was PHV + Years of Practice (BF₁₀ = 27.867), which provides very strong evidence in favor of simultaneously including biological maturation PHV and years of practice to explain the variability of Active Time (s). Strong support was also shown by the models PHV (BF₁₀ = 16.528), PHV + Jump Height + Years of Practice (BF₁₀ = 13.971), and Ball + PHV + Years of Practice (BF₁₀ = 12.292). Overall, the best models share PHV as a central component and often incorporate Years of Practice. The average R^2^ for the models was 0.335 (95% CrI = [0.158, 0.501]), suggesting a moderate fit with appreciable uncertainty. Sensitivity analysis including covariates with BF_incl> 1 (PHV) indicated an average R^2^ of 0.328 [0.158, 0.491], maintaining a model fit with a 95% CI that can vary from moderate to high.

**Table 4:** Comparison of Bayesian models with random effects per subject (top 5 of 64) for the variable active times (s).

	P(M)	P(M|data)	BFM	BF10	error %
Null model (incl. subject and random slopes)	0.016	0.005	0.302	1.000	
(PHV) + Years of practice	0.016	0.133	9.672	27.867	0.535
(PHV)	0.016	0.079	5.399	16.528	2.535
(PHV) + Jump height + Years of practice	0.016	0.067	4.505	13.971	0.378
Ball + (PHV) + Years of practice	0.016	0.059	3.929	12.292	1.257

P(M), prior model probability; P(M|data), posterior model probability; BFM, Bayes factor; for the model; BF10, Bayes Factor (Model 1 vs. Model 0); PHV, peak height velocity.

Table 5 indicated strong evidence for PHV (BF_incl = 7.709) while Years of Practice showed weak to moderate evidence (BF_incl = 1.617) in the analysis of evidence on effects. The remaining effects presented BF_incl < 1 (Ball, PAQ-A, Jump Height, Tournaments), suggesting a lack of support for their inclusion. The subsequent averaged analysis indicates that the intercept was stable (*M* = 625.103, SD = 37.763; 95% CrI = [548.680, 699.740]). Critically, PHV showed a credible negative effect (*M* = −91.010, SD = 38.278; 95% CrI = [−168.250, −16.862]), with an interval that excludes 0. In contrast, the intervals for the remaining predictors included 0, indicating uncertainty about their influence.

**Table 5:** Evidence of inclusion of effects (BFincl) and confidence intervals of the posterior summary averaged in the Bayesian ANOVA of repeated measures for the covariates and the factor explaining the variability of active times (s).

	95% CrI posterior	P(incl|data)	P(excl|data)	BFincl
Ball				
Green	[−86.45, 2.32]	0.311	0.689	0.451
Stand.	[−26.63, 85.11]			
(PHV)	[−168.25, −16.86]	0.115	7.709	7.709
PAQ-A	[−135.93, 217.70]	0.663	0.508	0.508
Jump height	[−44.75, 15.73]	0.656	0.524	0.524
Years of practice	[−86.06, 7.23]	0.382	1.617	1.617
Tournaments	[−11.27, 8.14]	0.715	0.399	0.399

CrI, credibility interval; P(incl|data), posterior inclusion probability; P(excl|data), posterior exclusion probability; BFinl, Bayes factor for inclusion; PHV, peak height velocity; PAQ-A, general physical activity level.

**Figure 4:**
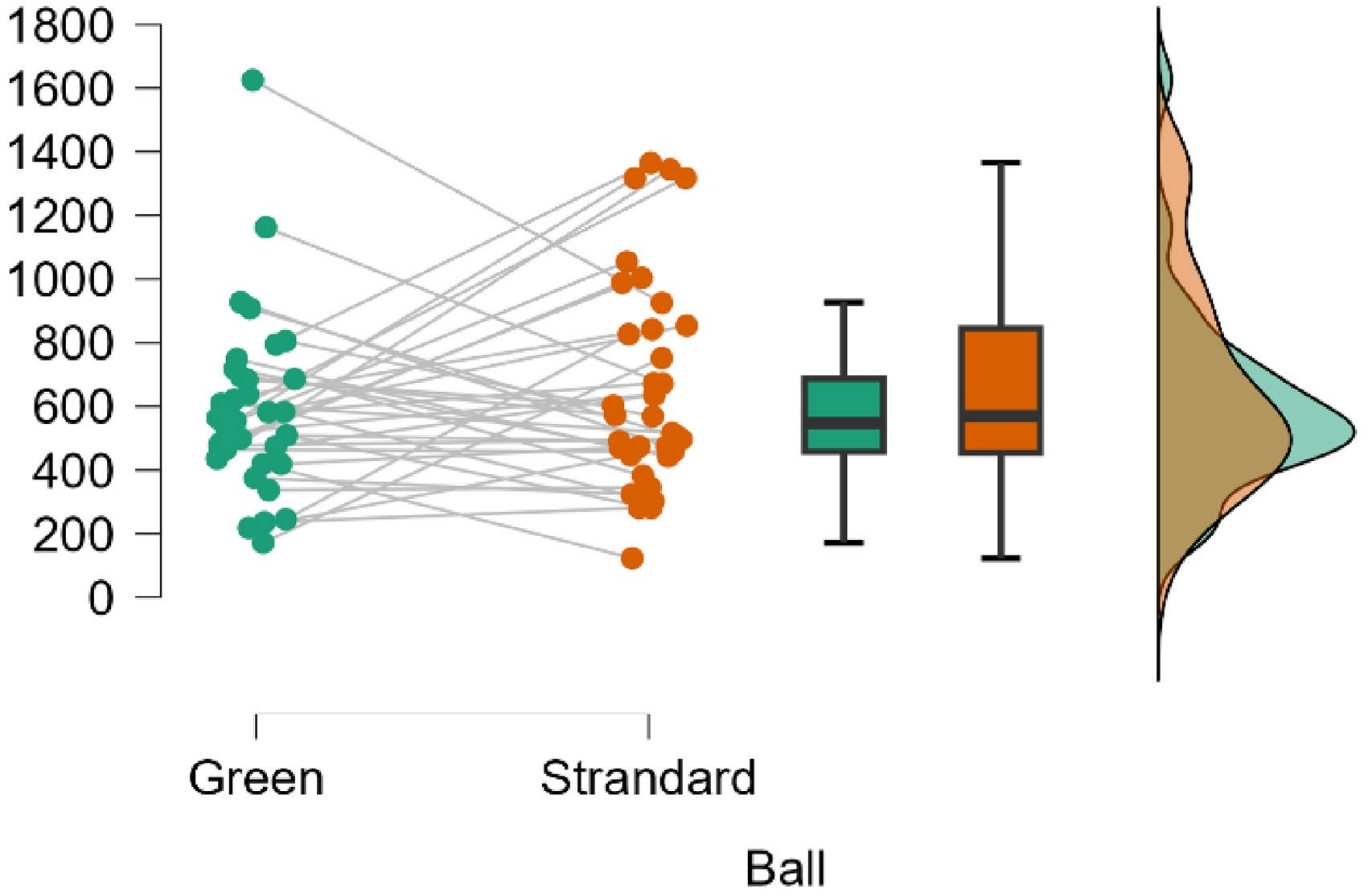
Comparison of the active time (s) variable according to the ball used in the matches.

The ball factor averages showed large areas of overlap, as can be seen in Figure 4: Green (*M* = 590.1, SD = 273.3; 95% CrI = [497.6, 682.6]) and Standard (*M* = 660.4, SD = 327.2; 95% CrI = [549.7, 771.1]). The comparison between Green vs. Standard showed equivalent posterior odds with a BF₁o = 0.302, implying greater support for the null hypothesis, which is approximately 3.31 times more likely than the alternative. The percentage error in the BF estimate was 0.038%.

### 3.3 Recoveries (s)

The Bayesian ANOVA of repeated measures with random effects per subject in Table 6 showed that the null model obtained the highest relative evidence (BF₁₀ = 1.000), while the best model with covariates was the one that included Years of Practice (BF₁₀ = 0.637). This value indicates that the data favor the null model over any alternative. The model-averaged R^2^ was 0.179, 95% CrI [0.101, 0.293].

**Table 6:** Comparison of Bayesian models with random effects per subject (top 5 of 64) for the variable Recoveries (s).

	P(M)	P(M|data)	BFM	BF10	error %
Null model (incl. subject and random slopes)	0.016	0.137	10.032	1.000	
Years of practice	0.016	0.087	6.038	0.637	0.739
Jump height	0.016	0.042	2.766	0.306	1.495
Tournaments	0.016	0.041	2.687	0.298	2.242
PAQ-A	0.016	0.040	2.593	0.288	1.252

P(M), prior model probability; P(M|data), posterior model probability; BFM, Bayes Factor; for the model; BF10, Bayes Factor (Model 1 vs. Model 0); PAQ-A, general physical activity level.

Table 7 on the inclusion effects analysis indicated that no predictor exceeded the threshold of evidence in favor of its inclusion. The credible intervals from the posterior summary averaged across models showed that none of the effects excluded zero, indicating considerable uncertainty in the parameters. In contrast, the intercept was stable (*M* = 1.004, 95% CrI [0.999, 1.007]).

**Table 7:** Evidence of inclusion of effects (BFincl) and confidence interval of the averaged posterior summary in the Bayesian ANOVA of repeated measures for the covariates and the factor explaining the variability of recoveries (s).

	95% CrI posterior	P(incl|data)	P(excl|data)	BF_incl_
Ball				
Green	[703.78, 856.87]	0.195	0.805	0.242
Stand.	[−58.74, 59.09]			
(PHV)	[−62.43, 57.66]	0.288	0.712	0.405
PAQ-A	[−67.82, 73.77]	0.298	0.702	0.424
Jump height	[−161.67, 130.69]	0.298	0.702	0.425
Years of practice	[−25.47, 33.40]	0.463	0.537	0.861
Tournaments	[−77.14, 15.32]	0.292	0.708	0.413

CrI, credibility interval; P(incl|data), posterior inclusion probability; P(excl|data), posterior exclusion probability; BFinl, Bayes factor for inclusion; PHV, peak height velocity; PAQ-A, general physical activity level.

**Figure 5:**
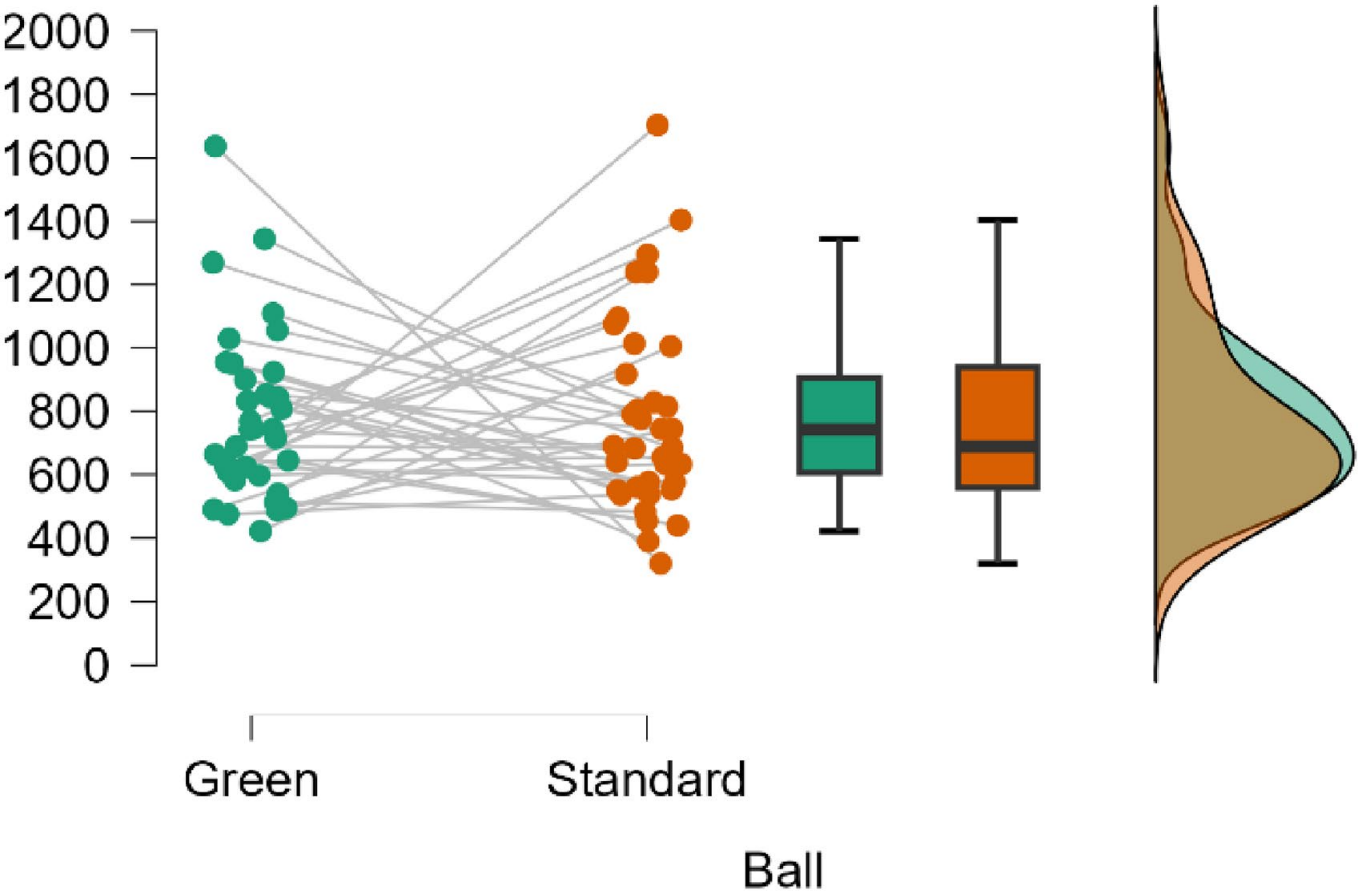
Comparison of the variable recoveries (s) according to the ball used in the matches.

Figure 5 shows very close mean values with broad overlaps: Green (*M* = 1.006, SD = 0.009, 95% CrI [1.003, 1.009]) and Standard (*M* = 1.002, SD = 0.006, 95% CrI [1.000, 1.004]). The pairwise analysis yielded a BF₁₀ = 2.096, reflecting anecdotal evidence in favor of the difference between conditions. The percentage error in the Bayesian factor estimate was practically zero (<0.001%).

### 3.4 Work-rest (s)

Table 8 shows the Bayesian ANOVA of repeated measures with random effects per subject and indicates that the best model was the one that included PHV (BF10 = 14.806), which indicates strong evidence in favor of including this covariate in the prediction of the dependent variable. Other models with substantial support were Ball + PHV (BF10 = 7.128) and PHV + Jump Height (BF10 = 6.978). The model-averaged R^2^ was 0.369, 95% CrI [0.202, 0.522], showing a high proportion of variance explained with a moderate range of uncertainty. Sensitivity analysis including covariates with BF_incl > 1 (PHV) indicated an average R^2^ of 0.358 [0.190, 0.511], maintaining a model fit with a 95% CrI that can vary from moderate to high.

**Table 8:** Comparison of Bayesian models with random effects per subject (top 5 of 64) for the work-rest variable (s).

	P(M)	P(M|data)	BF_M_	BF_10_	Error %
Null model (incl. subject and random slopes)	0.016	0.011	0.679	1.000	
(PHV)	0.016	0.158	11.814	14.806	1.793
Ball + (PHV)	0.016	0.076	5.184	7.128	2.897
(PHV) + Jump Height	0.016	0.074	5.065	6.978	0.874
(PHV) + Years of practice	0.016	0.055	3.666	5.156	1.052

P(M), Prior model probability; P(M|data), posterior model probability; BFM, Bayes Factor; for the model; BF10, Bayes Factor (Model 1 vs. Model 0); PHV, peak height velocity.

The inclusion effects analysis in Table 9 indicated that PHV presented the strongest evidence in favor of its inclusion (BF_incl = 9.761), reaching a strong level. The rest of the covariates (Ball, PAQ-A, Jump Height, Years of Practice, and Tournaments) suggest a lack of support for their inclusion, with BF_incl values <1. In addition, the model-averaged summary showed that only PHV had a credibility interval that excluded zero, indicating a credible negative effect (*M* = −0.126, DT = 0.052; 95% CrI [−0.234, −0.024]). The remaining effects (Ball, PAQ-A, Jump Height, Years of Practice, Tournaments) had 95% CrI that included zero, suggesting uncertainty about their influence. The intercept was stable (*M* = 0.848; 95% CrI [0.745, 0.951]).

**Table 9:** Evidence of inclusion of effects (BFincl) and confidence interval of the posterior mean fresumen in the Bayesian ANOVA of repeated measures for the variable.

	95% CrI posterior	P(incl|data)	P(excl|data)	BF_incl_
Ball				
Green	[−0.11, 0.02]	0.321	0.679	0.472
Standard	[−0.03, 0.10]			
(PHV)	[−0.23, −0.02]	0.907	0.093	9.761
PAQ-A	[−0.18, 0.24]	0.305	0.695	0.438
Jump height	[−0.05, 0.01]	0.361	0.639	0.565
Years of practice	[−0.07, 0.04]	0.294	0.706	0.416
Tournaments	[−0.01, 0.00]	0.291	0.709	0.410

CrI, credibility interval; P(incl|data), posterior inclusion probability; P(excl|data), posterior exclusion probability; BFinl, Bayes factor for inclusion; PHV, peak height velocity; PAQ-A, general physical activity level.

**Figure 6:**
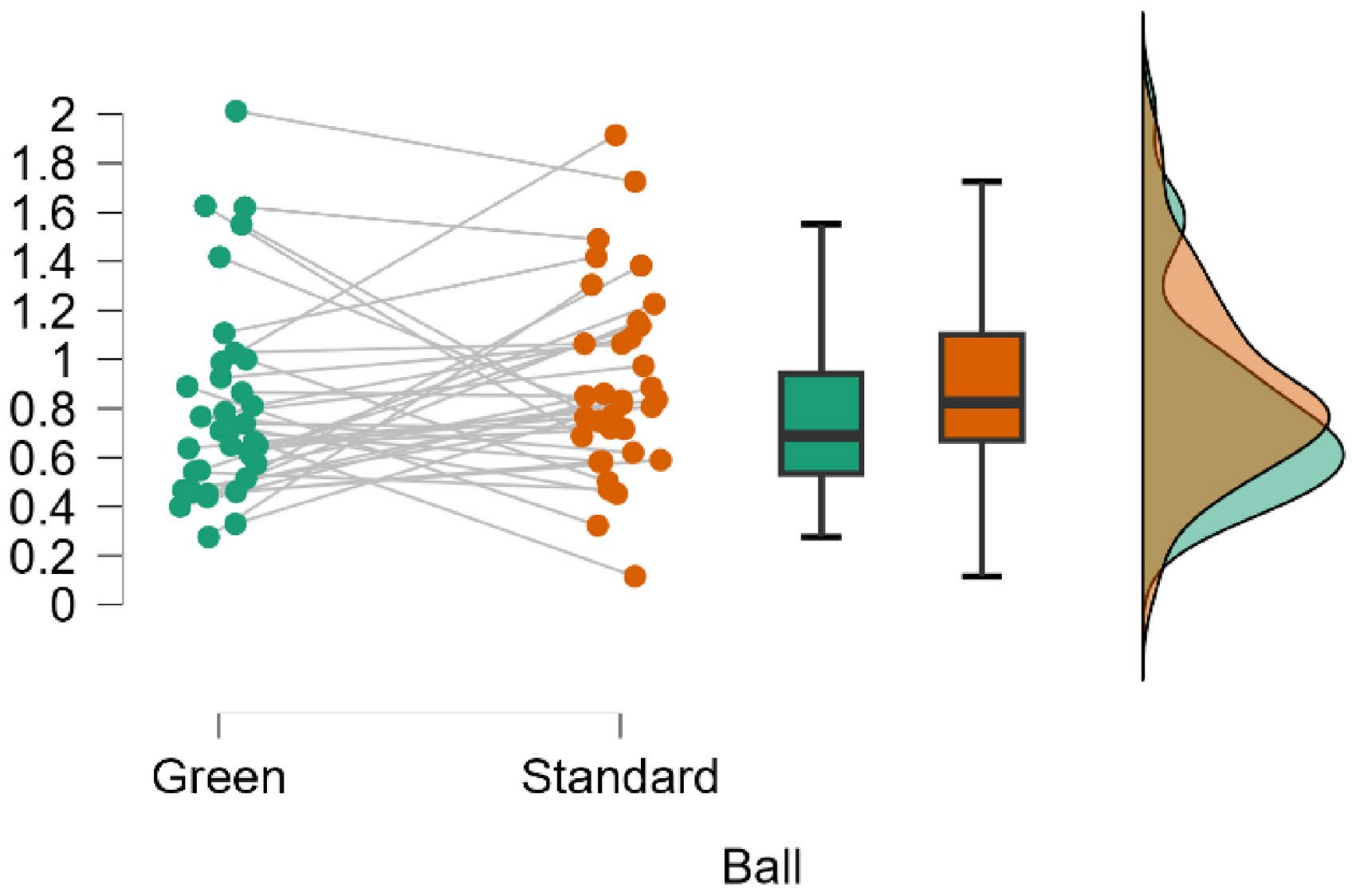
Comparison of the work-rest variable (s) according to the ball used in the matches.

Figure 6 shows similar mean values with broad overlaps in the credible intervals: Green (*M* = 0.802, SD = 0.404, 95% CrI [0.665, 0.939]) and Standard (*M* = 0.895, SD = 0.383, 95% CrI [0.765, 1.025]). Pairwise comparison showed a BF_10_ = 0.341, indicating that the data support the null hypothesis approximately 2.9 times more than the alternative. The percentage error in the Bayes factor estimate was low (<0.04%).

## 4 Discussion

This study aimed to quantify the effect of ball type on the external physical load of U10 tennis players, controlling for key individual covariates. The main finding reveals that, although the use of low-compression balls is a fundamental pedagogical principle for long-term athletic development (Farrow and Reid, 2010), its impact on the overall magnitude of external physical load in players at this stage was minimal. On the contrary, biological maturity, estimated by the PHV, is the most influential predictor of effort-pause, highlighting its role in modulating physical performance in formative stages.

In relation to the type of ball, our data show that the use of the green ball does not increase the physical load compared to the standard ball. This result is relevant, as previous studies had highlighted that low-compression balls modify the dynamics of the game, lengthening exchanges and increasing player participation (Larson and Guggenheimer, 2013; Kachel et al., 2015; Fitzpatrick et al., 2017, 2018). However, the findings of the present study suggest that, in the U10 category, these changes mainly affect the technical-tactical and pedagogical aspects (Buszard et al., 2016; Chapelle et al., 2023), without implying a significant increase in external load. This is positive, as it supports the regulations of the ITF and national federations (International Tennis Federation, 2023; RFET and CEAT, 2025), confirming that the green ball provides an adapted learning environment without substantially altering physical demands.

The literature has consistently established that adapted equipment, such as low-compression balls, modifies the characteristics of the game, resulting in longer rallies and a greater number of strokes per point (Schmidhofer et al., 2014; Kachel et al., 2015; Timmerman et al., 2015). This extension of the game would suggest an increase in cumulative active time. However, our data do not support this hypothesis. Although direct measures of internal load or pacing strategies were not analyzed, the consistency in external load values across different ball types may suggest a behavioral adjustment. It is plausible to hypothesize that young players unconsciously adapt their movement intensity to the specific affordances of the equipment to maintain a sustainable effort level. This aligns with the intermittent nature of tennis described by Crespo and Miley (1999), characterized by high-intensity efforts interspersed with recovery periods, where the total load is a product of both the duration and intensity of these efforts. However, future research incorporating physiological monitoring is needed to empirically confirm this ‘self-regulation’ mechanism.

On the other hand, PHV emerged as a strong predictor of active time (s) and work-rest (s), showing that players closer to their peak height growth tended to record less active time, contrary as expected. This finding coincides with what has been reported in the literature on the influence of maturation on the performance and conditional abilities of young tennis players (Kozieł and Malina, 2018; Fernandez-Fernandez et al., 2023). As these authors explain, young athletes approach and surpass their Peak Height Velocity, they undergo significant morphological and neural changes, including natural increases in muscle mass, stride length, and force-generating capacity. The practical interpretation is that, in stages of biological transition, economy of movement and neuromuscular adaptation can condition the load endured in competition, beyond the type of material used. Therefore, rather than the type of ball, it is the biological phase that conditions the physical demands in competition, which reinforces the need to individualize training and competitive planning according to the maturation profile.

In terms of experience, years of practice showed weak to moderate effects on some variables, suggesting that while continued exposure to training contributes to better coping with demands, its influence is less than that of maturity. For its part, the level of general physical activity did not show a clear association with the load, which could reinforce the idea that the specificity of tennis is more decisive than overall physical practice in explaining the demands of competition (Kowalski et al., 1997; Martínez-Gómez et al., 2009).

Overall, the results of this study confirm that adapting the type of ball in the U10 category does not substantially change the external physical load on players. This evidence is of great practical relevance, as it demonstrates that low-compression (green) balls do not increase the load, allowing coaches to focus on technical and tactical development. This approach is in line with the principles of individualization and sustainability in children’s sports (Soligard et al., 2016; Broadbent et al., 2021). Although the benefits of modified equipment are well known at the technical-tactical levels (Fitzpatrick et al., 2017, 2018), the contribution of our findings lies in the perspective of physical load and maturity status.

These results have important practical implications for coaches, suggesting that the green ball is an appropriate teaching tool regarding external load management. It allows for skill development while maintaining external load demands within manageable ranges. In addition, it highlights the need for coaches to consider maturity level rather than chronological age when planning workloads and content, tailoring training to each player’s biological profile.

### 4.1 Limitations and future directions

The present study has some limitations to consider for future analysis, such as the sample size. Although it is difficult to find competitive players U10 with a certain level of tennis, the sample is too small to generalize these results, even though the competition format was designed so that players could play as many matches as possible and none were eliminated. The distribution of participants was uneven regarding sex, and there was heterogeneity in the players’ experience levels. Given the ecological constraints and the resulting sample size, a Bayesian statistical approach was selected because is a particularly advantageous method in this context, as they do not rely on large-sample asymptotic theory to generate valid inferences, allowing for the quantification of uncertainty (Credible Intervals) without the strict requirements of frequentist power analysis. Future studies should aim for more balanced stratified samples to explicitly analyse potential sex-based or experience-based differences in physiological and behavioral responses.

Regarding the variables analyzed, it should be noted that only external load variables were analyzed, so internal load variables such as heart rate could be implemented to generate a more complete interpretation. It could also be complemented with technical-tactical analysis for a more integrated understanding of the game at these ages. In terms of the technology employed, while the 18 Hz GNSS units provide a high sampling rate, the specific context of small tennis courts may still induce some positional noise during rapid changes of direction. However, it is important to note that the primary external load indicators rely on inertial sensors (accelerometers) rather than satellite positioning and thus remain robust against GNSS positional error.

The simulated competition format does not fully reproduce real competition conditions, but at this age, almost every match was a challenge for them, and the players gave their best in each one. For future studies, it would be interesting to replicate this in real competitions, as well as during training sessions. Additionally, the use of the PAQ-A questionnaire presents a limitation. While valid for general populations in Spain, it may lack the sensitivity to discriminate between specific training loads in a cohort of already active competitive players (potential ceiling effect). Therefore, it should be interpreted as a broad indicator of general physical activity rather than a precise measure of athletic training volume.

Finally, in terms of practical application, it would be necessary to establish a series of guidelines and aspects to consider so that coaches know when to make the transition from green ball to standard ball, considering aspects of maturity rather than chronological age, so that the progression in their learning is much more appropriate.

## 5 Conclusion

This study demonstrates that the use of low-compression balls (green), compared to standard balls, does not significantly alter the magnitude of external physical load in U10 tennis players. This finding provides empirical support for current regulations, confirming that adapted equipment is a convenient teaching tool that allows coaches to focus on technical and tactical development without causing physical overload. On the contrary, biological maturity, estimated through PHV, emerges as the most decisive individual factor in modulating the physical demands of competition, directly influencing the effort-rest ratio of players. Therefore, the results underscore the need to prioritize individualized training based on maturity status over chronological age. For sports practice, this implies that load planning and the transition between training stages (including changing ball types) should be guided by the athlete’s biological profile to ensure safe, sustainable, and effective long-term development.

## Data Availability

The dataset is not publicly available due to ethical restrictions concerning research involving minors and institutional data protection policies approved by the Ethics Committee of the University of Valencia (approval code 2025-FIS-3843453). Requests to access the datasets should be directed to José María Giménez-Egido, josemaria.gimenez@um.es.
